# Risk factors and clinical outcomes in patients undergoing cytoreductive surgery with concomitant ureteric reimplantation

**DOI:** 10.1515/pp-2021-0130

**Published:** 2021-11-15

**Authors:** Anais Alonso, Shoma Barat, Helen Kennedy, Meredith Potter, Nayef Alzahrani, David Morris

**Affiliations:** Liver and Peritonectomy Unit, St George Hospital, Kogarah, Australia; St George and Sutherland Clinical School, University of New South Wales, Kogarah, Australia; College of Medicine, Al Imam Mohammad Ibn Saud Islamic University, Riyadh, Saudi Arabia

**Keywords:** cytoreductive surgery, peritoneal carcinomatosis, peritoneal surface malignancy, peritonectomy, ureteric reimplantation

## Abstract

**Objectives:**

There are currently scarce data exploring ureteric reimplantation (UR) during cytoreductive surgery (CRS).

**Methods:**

We identified patients undergoing CRS for peritoneal surface malignancies (PSM) of any origin at a single high-volume unit. UR was defined as ureteroureterostomy, transureterouretostomy, ureteroneocystostomy, ureterosigmoidostomy or ileal conduit performed during CRS. Peri-operative outcomes, long-term survival and risk factors for requiring UR were analysed.

**Results:**

Seven hundred and sixty-seven CRSs were identified. Twenty-three (3.0%) procedures involved UR. Bladder resection and colorectal cancer (CRC) were associated with increased risk of UR (bladder resection: OR 12.90, 95% CI 4.91–33.90, p<0.001; CRC: OR 2.51, 95% CI 1.05–6.01, p=0.038). UR did not increase the risk of Grade III–IV morbidity or mortality. The rate of ureteric leak was 3/23 (13.0%) in the UR group. Mean survival was equivocal in patients with CRC (58.14 vs. 34.25 months, p=0.441) but significantly lower in those with high-grade appendiceal mucinous neoplasm (HAMN) undergoing UR (73.98 vs. 30.90 months, p=0.029).

**Conclusions:**

UR during CRS does not increase major morbidity or mortality for carefully selected patients, and is associated with low rates of urologic complications. Whilst decreased survival was apparent in patients with HAMN undergoing UR, it is unclear whether this relationship is causal.

## Introduction

During cytoreductive surgery (CRS) for peritoneal surface malignancies (PSM), complete cytoreduction may necessitate multiple organ resections. Ureteric reimplantation (UR) may be indicated in cases of tumour invasion or iatrogenic injury, and is performed in 0.3–10.1% of CRSs [1], [2], [3], [4], [5], [6]. Ureteric obstruction has long been believed to contraindicate CRS due to evidence suggesting it reduces the ability to achieve complete cytoreduction [7, 8]. However, with the introduction of specialised high volume centres, this paradigm of thinking is shifting [9]. Resultantly, only one small (n=20) retrospective study by Morkavuk et al. [6] exists, which explores peri-operative and long-term outcomes in patients undergoing UR during CRS. Additionally, no study has examined risk factors for requiring UR during CRS. Investigation of such data is warranted to allow for appropriate selection of candidates for CRS and pre-operative counselling regarding expected outcomes. The aim of this study is to explore the risk factors and clinical outcomes in patients undergoing CRS with and without concomitant UR.

## Materials and methods

The research related to human use has complied with all the relevant national regulations, institutional policies, and in accordance with the tenets of the Helsinki Declaration, and has been approved by St George Hospital’s Human Research Ethics Committee (approval number 18/078). Data pertaining to all CRSs performed at St George Hospital, Sydney, Australia from January 1996 to present are collected in a prospectively maintained database. Selection of candidates for CRS, including pre-operative work-up and management, is described elsewhere by our unit [10]. CRS was performed using Sugarbaker’s technique [11], with or without intraperitoneal chemotherapy, as previously described by our unit [12]. Following CRS, patients were followed up at three-monthly intervals for the first 12 months and six-monthly intervals thereafter, until the last date of contact or death.

Patients were retrospectively identified for inclusion in this study if they underwent CRS for PSM of any origin with peritoneal cancer index (PCI) ≥1 between September 2009 (corresponding to the commencement of electronic medical records at St George Hospital) and December 2017 (to allow for sufficient follow-up for overall survival). Patients were excluded for missing data or if their operation report was unavailable electronically.

UR was defined as ureteroureterostomy, transureterouretostomy, ureteroneocystostomy, with or without psoas hitch or Boari flap, ureterosigmoidostomy or ileal conduit performed during CRS. Prior to anticipated UR, as deemed necessary during multidisciplinary team (MDT) discussion, routine ureteric stenting was performed by the urologic surgical team at out unit’s hospital, followed by UR performed by our CRS surgical team. In the case of intraoperative ureteric injury requiring reimplantation, ureteric stents were placed by our CRS team prior to performing UR. To identify patients undergoing UR, we searched our database using the keywords “ureter*”, “implant*”, “psoas hitch”, “Boari flap” and “ileal conduit”. All other patients were allocated to the non-UR group.

### Statistical analyses

The data were analysed by International Business Machines Corporation (IBM) Statistical Product and Service Solutions Statistics for Windows Version 26 (IBM, New York, United States). Data were analysed by procedure rather than patient, i.e. one patient may correspond to multiple entries. Organ resection was defined as either partial or total resection. Perioperative morbidity was categorised using the Clavien–Dindo classification of surgical complications [13], and included any such complication occurring during the same hospital admission as that for CRS. In-hospital mortality was defined as death during the same hospital admission as that for CRS.

Data were tested for normality using the Kolmogorov–Smirnov method. Between-group comparisons were undertaken with the Mann–Whitney U, independent-samples t-, Fisher’s exact and chi-square tests, as appropriate. To determine the odds ratios (OR) for risk factors for UR, binary logistic regression was performed. Variables with a probability value of <0.10 on univariate analysis were included in multivariate analysis. Survival analysis was performed using the Kaplan–Meier method. Survival was calculated from date of CRS to date of last contact or death, and was reported per patient not per procedure. Previous work by our group has demonstrated that survival following CRS is influenced by tumour origin [14], and thus overall survival was reported following stratification by tumour origin. Five-year survival rates only included patients undergoing CRS prior to 2016 to allow 5 years follow-up. A probability value of <0.05 was considered statistically significant.

## Results

Between September 2009 and December 2017 inclusive, 842 CRSs were performed by our unit. Of these, 75 (8.9%) procedures were excluded from analysis. This rendered 767 (91.1%) procedures, corresponding to 681 patients, eligible for inclusion. Of these, 23 patients underwent 23 (3.0%) URs, with no patient undergoing multiple URs in successive CRSs. Between patients who did and did not undergo UR, there were significant differences in the site of primary tumour and peri-operative chemotherapy regime. Patients in the UR group were more likely to undergo concomitant bladder resection. No patients with mesothelioma or low-grade appendiceal mucinous neoplasm underwent UR (Table 1).

**Table 1:** Patient demographics and clinical characteristics (n=767).

	No UR (n=744)	UR (n=23)	p-Value
Age, years, median (IQR)^a^	56 (46–65)	57 (47–62)	0.850
Sex, n (%)^b^			0.343
Male	333 (44.8)	8 (34.8)	
Female	441 (55.2)	15 (65.2)	
Diagnosis, n (%)^c^			0.012
CRC	235 (31.6)	13 (56.5)	
LAMN	150 (20.2)	0 (0.0)	
HAMN	223 (30.0)	7 (30.4)	
Mesothelioma	60 (8.1)	0 (0.0)	
Ovarian cancer	28 (3.8)	2 (8.7)	
Other	48 (6.5)	1 (4.3)	
Organ resection, n (%)			
Bladder^c^	26 (3.5)	8 (34.8)	<0.001
Colon^b^	501 (67.4)	18 (78.3)	0.274
Kidney^c^	14 (1.9)	2 (8.7)	0.080
Liver^c^	68 (9.1)	3 (13.0)	0.463
Small bowel^b^	363 (48.8)	12 (52.2)	0.749
Spleen^b^	283 (38.0)	7 (30.4)	0.459
Stomach^c^	70 (9.4)	4 (17.4)	0.267
Uterus/ovary^c,d^	181 (44.0)	5 (33.3)	0.412
PCI, median (IQR)^a^	14 (6–28)	8 (4–21)	0.083
Chemotherapy, n (%)^c^			0.022
EPIC	19 (2.6)	0 (0.0)	
HIPEC	529 (71.1)	22 (95.7)	
EPIC + HIPEC	168 (22.6)	0 (0.0)	
None	28 (3.8)	1 (4.3)	
Previous CRS, n (%)^b^	212 (28.5)	4 (17.4)	0.244

^a^Mann–Whitney U test, ^b^Chi-square test, ^c^Fisher’s exact test, ^d^Females only, n=456. CRC, colorectal cancer; EPIC, early postoperative intraperitoneal chemotherapy; HAMN, high-grade appendiceal mucinous neoplasm; HIPEC, hyperthermic intraperitoneal chemotherapy; IQR, interquartile range; LAMN, low-grade appendiceal mucinous neoplasm; PCI, peritoneal cancer index; UR, ureteric reimplantation.

### Ureteric involvement and reimplantation

Of those undergoing UR, 21/23 (91.3%) cases were indicated due to assumed tumour invasion, which was confirmed with histopathology in 14 patients. In 2/23 (8.7%) patients, UR was indicated due to iatrogenic ureteric injury. Of those undergoing UR, 20/23 (87.0%) patients underwent ureteroneocystostomy. Two (8.7%) of these procedures involved bilateral reimplantation. Additional procedures in this group included psoas hitch in 7/23 (30.4%), partial bladder resection in 2/23 (8.7%) and unilateral nephrectomy in 1/23 (4.3%). Three (13.0%) patients underwent ileal conduit formation, one (4.3%) of whom also underwent unilateral nephrectomy.

### Risk factors for requiring UR during CRS

On univariate analysis, concomitant urologic organ resection (bladder, kidney) and colorectal cancer (CRC) were predictive of requiring UR. On multivariate analysis, only bladder resection and CRC remained significant. Neither age, sex, PCI nor previous CRS were predictive of requiring UR (Table 2).

**Table 2:** Predictive factors for requiring UR (n=767).

	Univariate analysis		Multivariate analysis	
	OR (95% CI)	p-Value	OR (95% CI)	p-Value
Age	1.00 (0.97–1.03)	0.973	–	–
Female sex	1.52 (0.64–3.63)	0.346	–	–
Diagnosis				
CRC	2.82 (1.22–6.51)	0.016	2.51 (1.05–6.01)	0.038
HAMN	1.02 (0.42–2.52)	0.962	–	–
Organ resection				
Bladder	14.73 (5.74–37.82)	<0.001	12.90 (4.91–33.90)	<0.001
Colon	1.74 (0.64–4.74)	0.279	–	–
Kidney	4.97 (1.06–23.25)	0.042	2.53 (0.47–13.71)	0.282
Liver	1.49 (0.43–5.15)	0.527	–	–
Small bowel	1.15 (0.50–2.62)	0.749	–	–
Spleen	0.71 (0.29–1.75)	0.461	–	–
Stomach	2.03 (0.67–6.13)	0.210	–	–
Uterus/ovary^a^	0.64 (0.21–1.89)	0.415	–	–
PCI	0.97 (0.93–1.01)	0.111	–	–
Previous CRS	0.53 (0.18–1.57)	0.251	–	–

^a^Females only, n=441. CI, confidence interval; CRS, cytoreductive surgery; OR, odds ratio; PCI, peritoneal cancer index.

### Peri-operative outcomes

Patients undergoing UR required a significantly longer operating time and length of stay (LoS), but these were not associated with an increased length of intensive care unit admission, nor increased rates of perioperative Grade III–IV morbidity or mortality (Table 3).

**Table 3:** Peri-operative outcomes (n=767).

	No UR (n=744)	UR (n=23)	p-Value
Operating time, hours, median (IQR)^a^	8.05 (6.4–9.7)	9.7 (7.8–11.4)	0.003
CC score, n (%)^b^			0.202
0	546 (73.4)	21 (91.3)	
1	168 (22.6)	2 (8.7)	
2	29 (3.9)	0 (0.0)	
3	1 (0.1)	0 (0.0)	
Length of ICU admission, days, median (IQR)^a^	2 (1–3)	2 (2–3)	0.464
LoS, days, median (IQR)^a^	19 (14–28.75)	25 (20–43)	0.002
Grade III–IV morbidity, n (%)^c^	264 (35.5)	10 (43.5)	0.431
In-hospital mortality, n (%)^b^	14 (1.9)	1 (4.3)	0.369

^a^Mann–Whitney U test, ^b^Fisher’s exact test, ^c^Chi-square test. CC, completeness of cytoreduction; ICU, intensive care unit; IQR, interquartile range; LoS, length of stay; UR, ureteric reimplantation.

In the UR group, urologic complications included urinary tract infection (UTI) in 4/23 (17.4%), including urosepsis in 1/23 (4.3%), ureteric leak in 3/23 (13.0%), renal impairment in 2/23 (8.7%), including acute kidney injury (AKI) in 1/23 (4.3%) following cisplatin hyperthermic intraperitoneal chemotherapy (HIPEC), and vesicovaginal fistula in 1/23 (4.3%). In-hospital mortality occurred in 1/23 (4.3%) on day 19 post-CRS (cause of death unavailable).

In the non-UR group, urologic complications included UTI in 45/744 (6.0%), including urosepsis in 2/744 (0.3%), ureteric leak or injury in 14/744 (1.9%), though none of these required subsequent UR, renal impairment in 27/744 (3.6%), including AKI requiring dialysis in 1/744 (0.1%), vesicovaginal fistula in 1/744 (0.1%), urinary fistula in 1/744 (0.1%), urinary retention in 3/744 (0.4%), hydronephrosis in 3/744 (0.4%) and bladder injury in 1/744 (0.1%). Of the 15 (2.0%) in-hospital deaths, none were reported to be related to urologic complications.

### Survival

**Figure 1:**
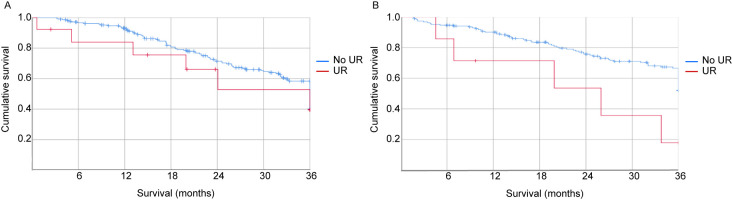
Overall survival. Patients undergoing cytoreductive surgery (CRS) for peritoneal surface malignancies of any origin at a single high-volume unit between September 2009 and December 2017 were retrospectively identified from a prospectively maintained database. Patients were divided into two groups based on whether they did or did not undergo ureteric reimplantation (UR) during CRS, which were further stratified by tumour origin. Survival was calculated from date of CRS to date of last contact or death, and was analysed using the Kaplan–Meier method. (A) Survival of patients with colorectal cancer (n=225), including 13 who underwent UR and 212 who did not. (B) Survival of patients with high-grade appendiceal mucinous neoplasm (n=193), including 7 who underwent UR and 186 who did not. UR, ureteric reimplantation

After stratifying by tumour origin, 418 patients with CRC and high-grade mucinous neoplasm (HAMN) were available for survival analysis. Of the 225 patients with CRC, 13 (5.8%) underwent UR and 212 (94.2%) did not. Of the 193 with HAMN, seven (3.6%) underwent UR and 186 (96.4%) did not. For 5-year survival analysis, 161 patients with CRC underwent CRS prior to 2016, including nine (5.6%) who underwent UR and 152 (94.4%) who did not, whilst 145 patients had HAMN, 6 (4.1%) of whom underwent UR and 139 (95.9%) of whom did not. Overall survival was significantly shorter in patients with HAMN who underwent UR, whilst the shorter survival for patients with colorectal cancer (CRC) undergoing UR was not statistically significant (Table 4, Figure 1).

**Table 4:** Overall survival.

	Survival, months		Survival, %		
	Mean (95% CI)	p-Value	1-year	3-year	5-year
CRC		0.441			
No UR	58.14 (50.81–65.47)		180/212 (84.9)	76/212 (35.8)	30/152 (19.7)
UR	34.35 (22.25–46.45)		10/13 (77.0)	4/13 (30.8)	0/9 (0.0)
HAMN		0.029			
No UR	73.98 (64.85–83.11)		157/186 (84.4)	87/186 (46.8)	39/139 (28.1)
UR	30.90 (9.52–52.27)		4/7 (57.1)	1/7 (14.3)	1/6 (16.7)

CI, confidence interval; CRC, colorectal cancer; HAMN, high-grade appendiceal mucinous neoplasm; UR, ureteric reimplantation.

## Discussion

In this study, UR was performed in 3.0% of patients undergoing CRS. This is consistent with published literature, which reports rates of 0.3–10.1% [1], [2], [3], [4], [5], [6]. Encouragingly, UR was not associated with increased risk of major morbidity or in-hospital mortality, demonstrating safety in patients following careful pre-operative selection. Owing to this, the increased LoS experienced by patients undergoing UR is likely of limited clinical significance apart from associated costs. Specifically, rates of urologic complications were also low, and similar to those reported during CRS with HIPEC and UR in the only other published study [6]. Of note, work by Pinar et al. [15] suggests that ureteroneocystostomy during CRS with HIPEC reduces the incidence of urinary fistula compared to ureteroureterostomy but has no effect in reducing major morbidity or mortality. In keeping with this, ureteroureterostomy is not routinely performed by our unit, and did not account for any of the URs performed in this study. Further investigations should examine other factors predisposing to urologic complications. Additionally, the perioperative mortality rate for patients undergoing UR in our cohort was notably lower than that reported by Morkavuk et al. [6] (4.3% vs. 10%) and promisingly, was consistent with published results of CRS, regardless of concomitant UR [16], [17], [18]. The lack of control patients in Morkavuk et al.’s study [6] makes it difficult to identify a cause for this discrepancy.

Previous work by our group has demonstrated that survival following CRS is influenced by tumour origin [14]. In our cohort, the distribution of diagnoses differed between the study and control groups, rendering the reporting of overall survival for the entire cohort inappropriate. The two groups were thus stratified by tumour origin, with diagnoses apart from CRC and HAMN excluded from analysis owing to small numbers in the UR group. This revealed significantly shorter survival in patients with HAMN undergoing UR, however, the retrospective nature of the study deems it difficult to determine if this relationship is a direct cause of the studied intervention, or whether differences between the two groups could better account for this, such as adjuvant chemotherapy or further surgical intervention, important factors influencing survival but outside the scope of our study. Prospective data may assist in clarifying this. In patients with CRC, survival was equivocal, regardless of whether UR was performed. It is possible that this lack of difference is due to inadequate power however the rarity of PSM renders it difficult to recruit larger sample sizes. It is important to note that the reported survival is likely underestimated, owing to the study’s retrospective design. Patients were not followed-up according to a strict protocol, resulting in a large loss to follow-up and survival that likely greatly exceeds reported values. Even so, the overall survival of the UR group is substantially longer than reported by Morkavuk et al. [6] (11.6 months), though mean follow-up was only 13.3 months in this study. In a systematic review of concomitant urologic interventions during CRS, no difference in overall survival was demonstrated in any of the four studies reporting this outcome [3], [4], [5, 19]. Recurrence-free survival would be of additional significance but unavailable from our database.

Bladder resection was identified as a risk factor for requiring UR. This can be easily explained as UR is commonly performed to facilitate bladder resection, or vice versa, and is thus likely of little clinical significance. Additionally, our results demonstrate increased likelihood of UR in patients with CRC compared to other PSM. The reason for this remains unclear but could include an increased tendency to have undergone previous abdominopelvic surgery or radiation, resulting in adhesions necessitating UR. This may also explain why 33.3% of cases of suspected ureteric invasion were overturned following histopathological assessment in the wider UR group. On the contrary, for patients with positive histopathology of the ureter, previous ureteric dissection may have disrupted surgical planes, allowing for a new pathway of invasion. It is also important to note that tumour burden did not appear to be higher in patients with UR, with the PCI and extent of abdominopelvic organ resection no greater in the intervention group compared to controls. Contrarily, Leapman et al. [4] have reported an increased risk of urologic reconstruction in patients with extensive organ involvement; however, this result is not specific to UR.

Ureteric obstruction has long been believed to contraindicate CRS due to evidence suggesting it reduces the ability to achieve complete cytoreduction [7, 8]. In our cohort, a completeness of cytoreduction (CC) score of zero was achieved more frequently in patients undergoing UR than those who did not, though not to a significant extent. This is reflected in more recent literature [3, 19], which has also demonstrated a lack of difference in CC scores between patients who did and did not require urologic intervention during CRS. Additionally, our data indicated a non-significantly reduced PCI in patients undergoing UR, suggesting that ureteric involvement is not associated with a more extensive disease profile that may make complete cytoreduction more difficult. However, the rate of ureteric obstruction in our cohort is unknown. It is also important to note that the results from this study only take into account patients considered suitable surgical candidates following extensive pre-operative assessment. Whilst our unit does not consider bladder or ureteric invasion in and of itself a contraindication provided complete cytoreduction is achievable, the characteristics of patients deemed unsuitable for resection are unknown, and may possibly contain a greater proportion of patients who may have required UR had CRS proceeded. This unknown degree of selection bias reinforces that the results of our study are not generalisable to all patients with PSM, only to those deemed suitable for cytoreduction following rigorous pre-operative MDT assessment.

This study is strengthened by its large sample size, derived from our high-volume unit that has performed over 1,500 CRSs since its induction. Contrarily, the retrospective nature of this study brings inherent limitations, with several important variables not recorded, including pre-operative malnutrition, a well-known risk-factor for post-operative morbidity and increased LoS [20, 21], and postoperative urinary dysfunction, a crucial quality-of-life consideration.

In summary, UR during CRS does not increase major morbidity or in-hospital mortality in carefully selected patients with PSM. UR is associated with low rates of urologic complications but significantly increased operating time and LoS. Whilst decreased long-term survival was apparent in patients with HAMN undergoing UR, it is unclear whether this relationship is causal. Long-term survival was equivocal in patient with CRC, regardless of whether UR was performed.
